# Impaired myocardial perfusion in rheumatoid arthritis is associated with impaired strain, strain rate, disease activity and myocardial oedema: a cardiovascular magnetic resonance study

**DOI:** 10.1186/1532-429X-17-S1-Q65

**Published:** 2015-02-03

**Authors:** Ntobeko A Ntusi, Emily Sever, Joseph Lockey, Jane M Francis, Stefan K Piechnik, Vanessa M Ferreira, Paul M Matthews, Paul B Wordsworth, Stefan Neubauer, Theodoros D Karamitsos

**Affiliations:** 1 OCMR, Division of Cardiovascular Medicine, University of Oxford, Oxford, UK; 2 Division of Cardiology, Department of Medicine, University of Cape Town, Cape Town, South Africa; 3 Division of Brain Sciences, Imperial College London, London, UK; 4 Bortnar Institute, Nuffield Department of Orthopaedics, Rheumatology and Musculoskeletal Sciences, University of Oxford, Oxford, UK

## Background

Rheumatoid arthritis (RA) commonly involves the cardiovascular system, and is associated with significant morbidity and mortality. Mechanisms of cardiovascular disease (CVD) involvement are not fully understood, but cardiovascular inflammation is thought to drive many of the CVD manifestations, including myocardial ischaemia. The clinical utility of CMR first-pass perfusion for assessment of myocardial ischaemia is well-established. The aim of this study was to assess whether RA patients without known epicardial coronary artery disease have evidence of myocardial hypoperfusion.

## Methods

55 RA patients (39 female, mean age 54 ± 11 years) with 55 matched controls (39 female, mean age 53 ± 10 years) were enrolled into the study. All patients with known cardiovascular disease were excluded. Study participants underwent CMR at 1.5T and the assessments included cine, tagging, T1 mapping, T2-weighted, perfusion, late gadolinium (0.15mmol/kg gadoteric acid - Dotarem®) imaging and ECV quantification. Comorbid status, disease activity index (DAS28-CRP) and duration of disease were recorded for each subject.

## Results

RA patients and controls were well matched for age, sex and comorbidities (Table 1). There was no significant difference in LV size, mass and ejection fraction between RA patients and controls (Table 2). Peak systolic circumferential strain and peak diastolic strain rate were impaired in patients. Myocardial perfusion reserve index was 1.5 ± 0.3 and 1.9 ± 0.4 (p<0.001) in RA and controls, respectively. Non-segmental (circumferential) subendocardial perfusion defects were seen in 47% and none (p<0.001) of RA patients and controls studied. Impaired MPRI correlated with peak systolic strain (R -0.71, p<0.001) and peak diastolic strain rate (R 0.63, p<0.001) in RA (Figure 1). Further, abnormal MPRI was associated with DAS28-CRP (R -0.38, p=0.005) and volume fraction of T2 SI ratio (R -0.29, p=0.036) in RA.

**Table 1:** Baseline characteristics of RA patients and controls

	Controls N=55	RA N=55	P value
Female sex, n (%)	39 (71)	39 (71)	1.00

Age, years	53 ± 10	54 ± 11	0.70

Hypertension, n(%)	4 (7)	9 (16)	0.24

Diabetes, n(%)	0	3 (5)	-

Hyperlipidaemia, n(%)	11 (20)	9 (16)	0.75

BMI, kg/m2	24 ± 4	26 ± 4	0.06

Methotrexate, n(%)	N/A	50 (91)	-

Chloroquine, n(%)	N/A	30 (55)	-

Leflunomide, n(%)	N/A	10 (18)	-

Sulfasalazine, n(%)	N/A	8 (15)	-

Rituximab, n(%)	N/A	3 (5)	-

Prednisolone, n(%)	N/A	5 (9)	-

DAS28-CRP	N/A	3.4 ± 1.4	-

ESR, mm/hr (median, IQR)	N/A	13 (8-17)	-

CRP, mg/L (median, IQR)	1 (1-2)	10 (5-16)	<0.001

Duration of RA, years (median, IQR)	N/A	9 (5-13)	-

Duration of DMARDs, years (median, IQR)	N/A	6 (3-8)	-

RF positive, n (%)	N/A	45 (82)	-

ACCP positive, n (%)	N/A	38 (69)	-

Continuous data are mean ± SD unless otherwise indicated.

Categorical data are frequency (percent) unless otherwise indicated.

ACCP, anti-cyclic citrullinated peptide antibodies; BMI, body mass index; CRP, C-reactive protein; DAS28-CRP, rheumatoid arthritis disease activity index incorporating 28 tender and swollen joint count and serum CRP; DMARD, disease modifying anti-rheumatic drug(s); ESR, erythrocyte sedimentation rate; IQR, interquartile range; RA, rheumatoid arthritis; RF, rheumatoid factor

**Table 2:** Myocardial structure, function and perfusion in RA patients and controls

	Controls N=55	RA N=55	P value
LVEDV indexed to BSA, ml/m2	78 ± 15	79 ± 15	0.92

LVESV indexed to BSA, ml/m2	22 ± 14	22 ± 8	0.96

LVEF, %	74 ± 4	72 ± 7	0.45

LV Mass indexed to BSA, g/m2	54 ± 11	55 ± 11	0.64

LA size, mm	27 ± 5	32 ± 6	<0.001

Mid SA circumferential strain	-18.7 ± 1.2	-17.0 ± 1.1	<0.001

Peak diastolic circumferential strain rate (s-1)	115 ± 21	82 ± 19	<0.001

Presence of LGE (%)	0	27 (49)	-

Volume fraction of LGE>2SD (%)	0	3.8 ± 0.3	-

Global myocardial T2 SI Ratio	1.5 ± 0.1	1.7 ± 0.3	<0.001

Volume fraction of oedema by T2 (%)	0	20 (8-33)	-

Average myocardial T1, ms	958 ± 26	971 ± 23	<0.001

Volume fraction of T1>990ms (%)	1 (1-6)	32 (18-49)	<0.001

ECV (%)	27.4 ± 2.9	30.1 ± 3.2	<0.001

Rest RPP	8, 982 ± 1, 716	8, 807 ± 1, 347	0.13

Stress RPP	11, 360 ± 1, 835	12, 530 ± 2, 547	<0.001

MPRI	1.9 ± 0.4	1.5 ± 0.3	<0.001

Proportion of non-segmental perfusion defects (%)	0	27 (49)	-

Continuous data are mean ± SD unless otherwise indicated.

ECV, extracellular volume; LA, left atrium; LGE, late gadolinium enhancement; LV, left ventricle/ventricular; LVEDV, left ventricular end-diastolic volume; LVEF, left ventricular ejection fraction; LVESV, left ventricular end-systolic volume; MPRI, myocardial perfusion reserve index; RA, rheumatoid arthritis; RPP, rate pressure product; SA, short axis; SI, signal intensity

## Conclusions

Myocardial perfusion is impaired in about half of asymptomatic RA patients with apparently normal hearts likely due to microvascular dysfunction. Abnormal perfusion reserve correlates with myocardial strain and RA disease activity.

## Funding

This study was funded by investigator-led grants from Guerbet and GlaxoSmithKline.

